# Differences in cervical cancer screening between immigrants and nonimmigrants in Norway: a primary healthcare register-based study

**DOI:** 10.1097/CEJ.0000000000000311

**Published:** 2017-10-05

**Authors:** Kathy A. Møen, Bernadette Kumar, Samera Qureshi, Esperanza Diaz

**Affiliations:** a Department of Global Public Health and Primary Care, University of Bergen; b Norwegian Center for Minority Health Research; c Department for Health and Society, University of Oslo, Oslo, Norway

**Keywords:** cancer screening, emigrants and immigrants, population register, primary healthcare, uterine cervical neoplasms

## Abstract

The prevalence of cervical cancer is high among some groups of immigrants. Although there is evidence of low participation in cervical cancer screening programs among immigrants, studies have been subject to selection bias and accounted for few immigrant groups. The aim of this study was to compare the proportion of several groups of immigrants versus nonimmigrants attending the cervical cancer-screening program in Norway. In addition, we aimed to study predictors for attendance to the screening program. Register-based study using merged data from four national registries. All Norwegian-born women (1 168 832) and immigrant women (152 800) of screening age for cervical cancer (25–69 years) registered in Norway in 2008 were included. We grouped the immigrants by world’s geographic region and carried out descriptive analyses and constructed several logistic regression models. The main outcome variable was whether the woman was registered with a Pap smear in 2008 or not. Immigrants had lower rates of participation compared with Norwegian-born women; Western Europe [adjusted odds ratio (OR), 95% confidence interval (CI): 0.84, 0.81–0.88], Eastern Europe (OR 0.64, 95% CI: 0.60–0.67), Asia (OR 0.74, 95% CI: 0.71–0.77), Africa (OR 0.61, 95% CI: 0.56–0.67) and South America (OR 0.87, 95% CI: 0.79–0.96). Younger age, higher income, residence in rural areas, and having a female general practitioner (GP) were associated with Pap smear. Longer residential time in Norway and having a nonimmigrant GP were associated with screening for some immigrant groups. Appropriate interventions targeting both immigrants and GPs need to be developed and evaluated.

## Abstract 2

Supplemental Digital Content is available in the text.

## Introduction

Cervical cancer is one of the few preventable cancers if detected early. It is the third most common cancer and the fourth most frequent cause of cancer deaths in women worldwide (Jemal *et al.*, 2011). However, cervical cancer prevalence and mortality are not evenly distributed. More than 85% of the cases and deaths occur in low-income and middle-income countries (Ferlay *et al.*, 2013). Cervix cancer is slightly more common in some immigrant groups living in Western countries than in the general population (Arnold *et al.*, 2010; Azerkan *et al.*, 2012).

The main factor for the development of cervical cancer is persistent infection with high-risk human papilloma virus. Many Western countries use the Papanicolaou stain (Pap smear) for cervical cancer screening (CCS). Several international studies show that immigrants have lower participation rates in preventive screening (Woltman and Newbold, 2007; Johnson *et al.*, 2008; Lofters *et al.*, 2010; Grandahl *et al.*, 2012; Berens *et al.*, 2014; Campari *et al.*, 2015; Ghebre *et al.*, 2015; Lee *et al.*, 2015) and when they eventually see a doctor, they are often diagnosed with severe forms of cervical cancer (Schleicher, 2007). However, these studies are often subject to selection bias, limited to one immigrant group or ethnic group, and rely on self-reported data.

Nearly 16% of the population in Norway was of migrant origin at the beginning of 2016 (Statistics Norway, 2016). In Norway, today, all women between 25 and 69 years receive a letter in Norwegian at 3-year intervals, inviting them to make an appointment with their general practitioner (GP) to take a Pap smear. Although the general attendance to this program has been 74% after reminders (Skare and Lönnberg, 2015), over half of the women diagnosed with cervical cancer have rarely or never taken a Pap smear (Cancer Registry of Norway, 2016). The proportion of women with immigrant background who attend this program is currently unknown.

Our hypothesis was that immigrants in Norway had lower but different attendance rates of CCS depending on their region of origin. In addition, we hypothesized that not only the characteristics of the women but also those of their GPs could influence women’s attendance to CCS. We took advantage of a nationwide multiregister study including information on all women registered in Norway and their GPs. Our aim was to compare the proportion of different groups of immigrants with nonimmigrant women registered by their GPs as having taken a Pap smear in 2008 and to study predictors for attendance to the CCS program for the different immigrant groups.

## Participants and methods

This was a cross-sectional study using merged data from four nationwide registries in Norway: The National Population Registry, the Norwegian Health Economics Administration Database (HELFO), the GPs’ database, and the 2008 Medical Birth Registry.

All Norwegian citizens and legal immigrants residing in Norway for over 6 months have a unique personal identification number and this was used to link the four registries. All legally registered immigrants are members of the National Insurance Scheme, which entitles them access to a GP and Emergency Primary Care services. All nonimmigrant women with both parents from Norway (1 168 832) and immigrant women defined as born abroad with both parents from abroad (152 800) in the age group for CCS (25–69 years) registered in Norway in 2008 were included in the study.

From the National Population Registry, we obtained information on study women in terms of age, immigration category (nonimmigrant or immigrant), reason for migration (refugee, work, family reunification, and other), length of stay in Norway (up to 2 years and longer than 2 years), municipal centrality (urban or rural), civil status (married, unmarried, and other – including widowed, divorced, separated, and others), education level (none, low: lower secondary school, middle: upper secondary school, and high: university/college), and personal annual income in Norwegian Kroners (NOK) (low: below 200 000 NOK, medium, and high: over 400 000 NOK). Immigrant’s country of origin was categorized by regions as follows: (i) Nordic countries, (ii) North America and Western Europe, (iii) Eastern Europe, (iv) Asia, (v) Africa, and (vi) South and Central America. As preliminary analyses showed similar results for Nordic countries and Western Europe/North America and for comparison with other studies, we regrouped these two regions into one called ‘Western Europe’.

HELFO data (HELFO, Tønsberg, Norway) were based on administrative claims registered from all patient contacts within the primary healthcare, including both consultations with GPs and Emergency Primary Care services. Diagnoses were based on the International Classification of Primary Care, version 2 (ICPC-2). For our study, we selected consultations with diagnoses related to screening for cervical cancer. The diagnoses included were X85 disease in cervix IKA, X86 abnormal cervical cytology, A981 cytology cervical screening, and 37 histological/cytological test and other gynecological illnesses. We created a binary variable as the main outcome variable, being ‘1’ for women with at least one of these diagnoses and ‘0’ for the rest of the women.

We obtained information from the Medical Birth Registry on whether the woman had given birth or not in 2008. From the GPs’ database, we obtained information on sex and immigrant background of the women’s GP.

This study is part of the project ‘Immigrants’ Health in Norway’, approved by the Regional Committee for Medical and Health Research Ethics and the Norwegian Data Inspectorate.

### Statistical analyses

We performed comparisons of demographic characteristics for nonimmigrants and immigrants using *χ*^2^ and analysis of variance for categorical and continuous variables, respectively. In addition, we compared the demographic characteristics of women with and without a Pap smear test for each of the regions of origin.

Binary logistic regression analyses were carried out with ‘being registered with a Pap smear test in 2008’ as the dependent variable. Our main explanatory variable was the patients’ region of origin, with nonimmigrants as the reference group. Other explanatory variables included the woman’s age, income, marital status, municipal centrality, pregnancy, and GP’s characteristics. We constructed several logistic regression models. First, we included each of the explanatory variables one by one. Model 1 included age categorized into three intervals in addition to region of origin. Model 2 added other socioeconomic variables: marital status, income, and municipality’s centrality to model 1. Model 3 further included GP’s sex and immigrant background. We used pregnancy in the preliminary analyses, but did not include it afterwards as the inclusion of this variable did not further improve the model measured by the Nagelkerke *R*^2^ value.

Finally, to explore effect modifications between region of origin and the other explanatory variables, we performed binary logistic regression of model 3 by region of origin.

We used SPSS 22.0 software package for statistical analyses. (SPSS - Statistical package for social sciences), IBM Corp. 2013. Armonk, New York, USA).

## Results

A total of 1 321 632 women with a mean age of 47.1 years (SD 12.6) were included in our study. Table 1 describes the sociodemographic characteristics of the study population by regional groups.

**Table 1:**
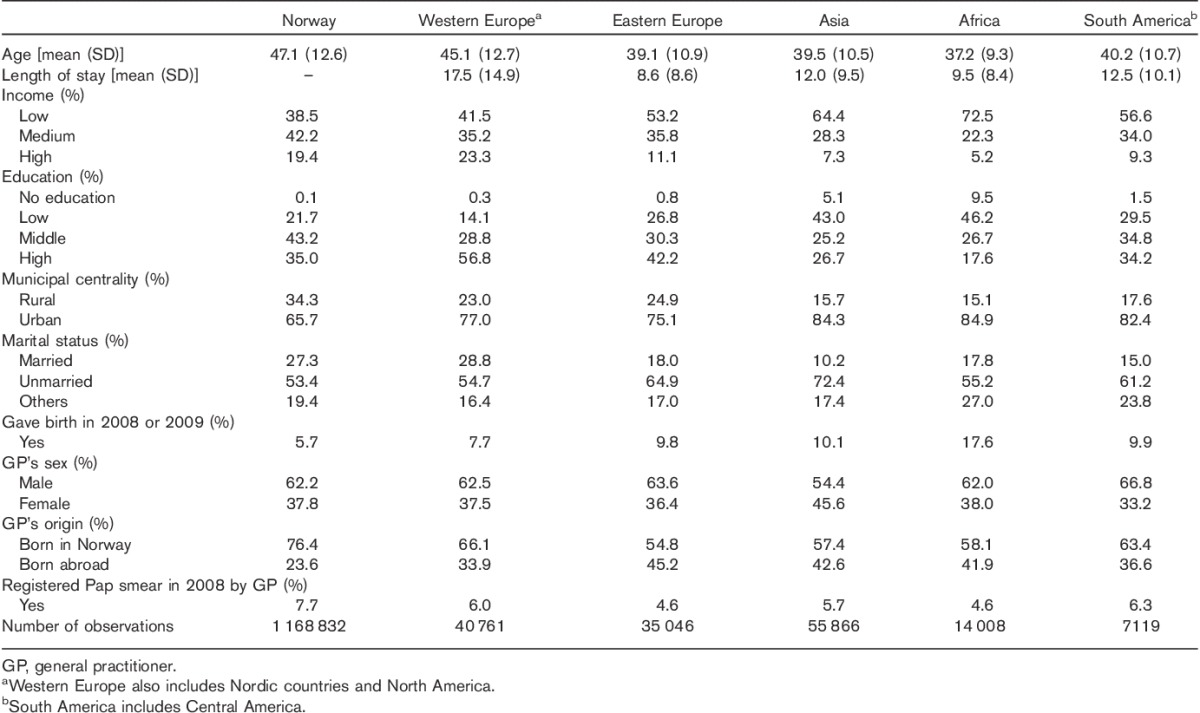
Sociodemographic characteristics of the study population by world regions

Immigrants had lived in Norway from 8 to 18 years. Compared with nonimmigrants, immigrant women were younger and more often lived in urban areas. Women from Western Europe had the highest income and education levels, whereas more than half of the women from Africa had either low or no reported education and had the lowest income levels. Women from Eastern Europe, Asia, and South America were often unmarried. A higher percentage of immigrants had been pregnant in 2008. Those from Asia more often had female GPs. Immigrants more often had GPs born outside Norway. Of the total 7.4% Pap smear registered in 2008, the highest registration was made among nonimmigrant women (7.7%) and the lowest among immigrant women from Africa and Eastern Europe (4.6%).

Demographic characteristics for women, both with and without Pap smear by region of origin, are presented as Supplementary data (Table S1), Supplemental digital content 1, *http://links.lww.com/EJCP/A118*. For both immigrants and nonimmigrants, younger women, with higher income, in rural areas and those who had not been pregnant were among those who took Pap smear more often. Among immigrants, no significant differences in taking Pap smears were observed by length of stay. Generally, women with female GPs had more Pap smears registered. The proportion of women with a Pap smear was significantly lower among women with an immigrant GP, except for women from Africa.

Table 2 shows the results from logistic regression analyses. Immigrants from all regions had a significantly lower probability of having a Pap smear registered compared with nonimmigrants in all models. Increasing age was associated negatively with Pap smear rates. Higher income, living in rural areas, having a female GP, and a Norwegian GP were associated significantly with more Pap smears in multivariate models. Although being married was associated with a Pap smear test in univariate analyses, the opposite was true in the adjusted models.

**Table 2:**
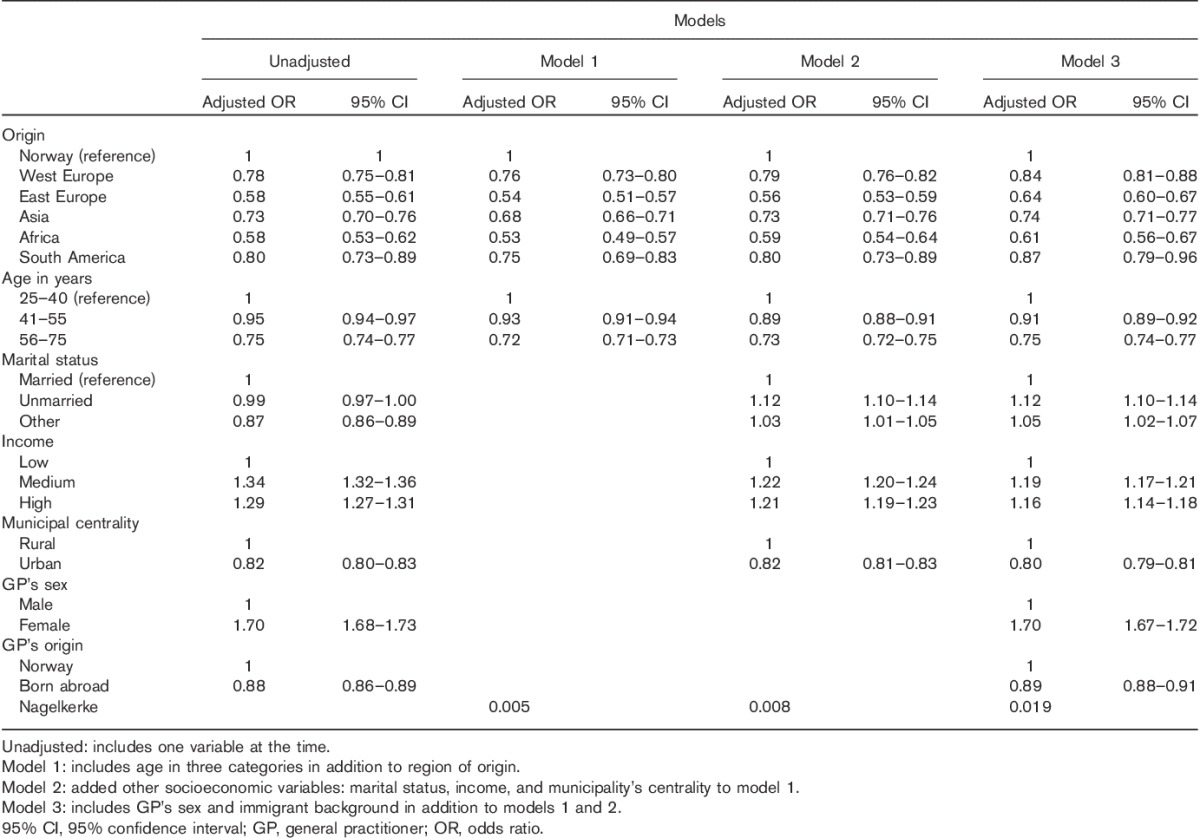
Binary logistic regression. Associations between Pap-smear attendance and immigrant background

Table 3 shows the adjusted logistic regression analyses for immigrant women by region of origin. The associations between screening and socioeconomic variables were in the same direction as for the population as a whole in terms of income and living in rural areas, but differed slightly for the various immigrant groups for other characteristics. Younger age was associated significantly with Pap smear for women from Eastern Europe and the age pattern seemed to be different for women from Asia, where women aged 41–55 years took the test significantly more often. The effect of length of stay in Norway on screening varied with the immigrant group, being positively associated for women from Eastern Europe, whereas most other groups had significantly lower attendance after 2 years. Being single was positively associated for women from Eastern Europe, Asia, and South America, whereas being married was associated with lower rates of Pap smears for women from Asia and South America. In terms of GP’s characteristics, having a female GP significantly increased the probability of taking a Pap smear for all groups, whereas having a GP born outside Norway was associated with significantly lower rates of Pap smear for Europeans and Asians, but not for women from Africa and South America.

**Table 3:**
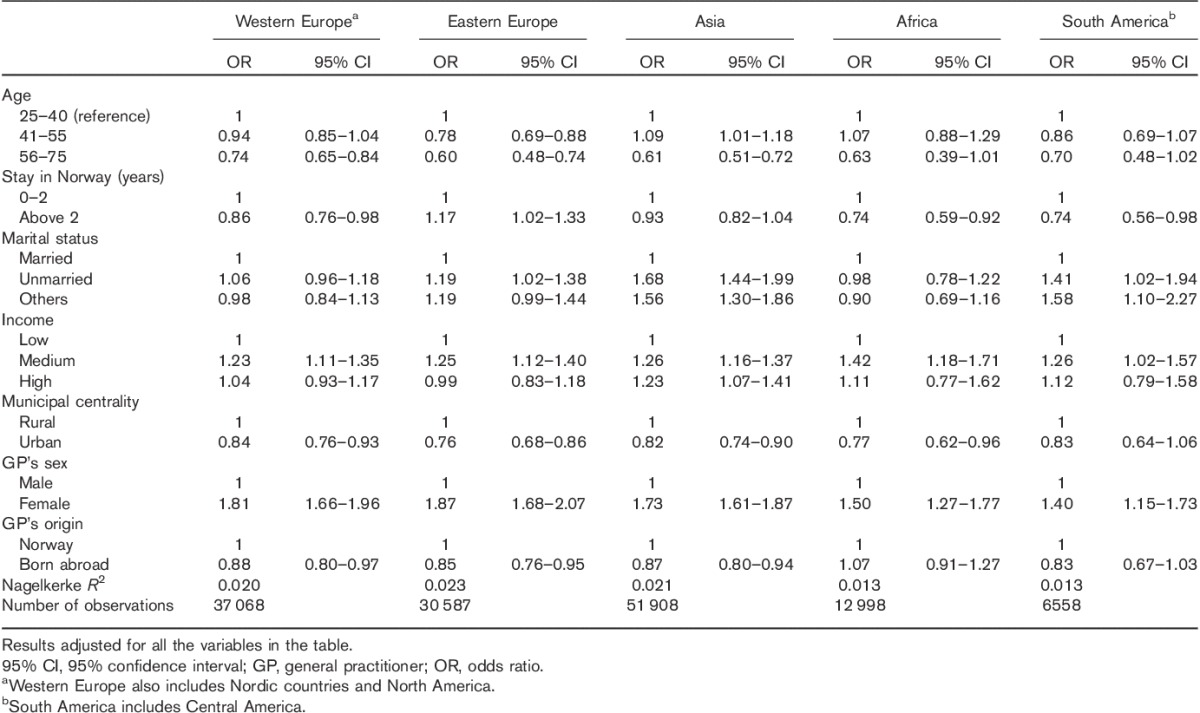
Binary logistic regression. Pap-smear attendance for immigrant women by region of origin

## Discussion

Our study confirms lower rates of participation in the preventive CCS program in Norway among immigrants compared with nonimmigrants. Higher income, residence in rural areas, and having a female GP were associated positively with Pap smear for both immigrants and nonimmigrants. Younger age was associated with Pap smears for nonimmigrants and most immigrant groups. Longer stay in Norway was significantly positively associated with higher attendance for women from Eastern Europe, but not for other immigrants. Having a Norwegian-born doctor was positively associated with screening for women from Western and Eastern Europe and Asia, but not for women from Africa or South America.

Our findings are in agreement with several international studies that report lower rates of CCS for immigrants (Woltman and Newbold, 2007; Lofters *et al.*, 2010; Berens *et al.*, 2014; Campari *et al.*, 2015; Ghebre *et al.*, 2015; Lee *et al.*, 2015), but with wide variations in screening by ethnic background (McDonald and Kennedy, 2007). In our study, women from Africa and Eastern Europe had the lowest rates of participation in CCS. Given the nature of our study, we cannot provide explanations for this finding, but several barriers described earlier could contribute toward explaining our results. We group these barriers into individual (including cultural, economic, and life situation related) and structural.

Cultural barriers mentioned in other studies include the belief that the healthcare system is for treatment not for prevention (Akers *et al.*, 2007), embarrassment, and the fear that screening threatens virginity (Coughlin *et al.*, 2006; Akers *et al.*, 2007). These barriers might, however, influence immigrants differentially. Embarrassment regarding circumcision, for example, can be especially important for women from Somalia (Lofters *et al.*, 2011; Shelton *et al.*, 2012; Ekechi *et al.*, 2014; Harcourt *et al.*, 2014), who represent the main group among women from Africa in our study.

However, culture and beliefs are not static, and acculturation tends to increase with longer stay in the new country. Although several studies describe a positive association between longer stay in the host country and Pap smear (McPhee *et al.*, 1997; Lofters *et al.*, 2011), other studies find that disparities in CCS attendance persist despite longer stay in the host country (Echeverria and Carrasquillo, 2006). In our study, length of stay in Norway was positively associated with screening for women from Eastern Europe, but negatively associated for women from Western Europe, Africa, and South America, despite different cut-offs of length of stay used in the analyses (Supplementary Table 2, Supplemental digital content 2, *http://links.lww.com/EJCP/A119*). This indicates an effect modification between length of stay and attendance for the different immigrant groups. Women from Poland represent the majority of immigrant women from Eastern Europe. A possible explanation for the association between length of stay and Pap smear for Eastern Europeans could be that these women prefer direct access to specialist healthcare as in their home countries compared with gatekeeping by GPs in Norway and might therefore travel to their own country to receive healthcare services during the first years in Norway (Lamkaddem *et al.*, 2012).

Economical barriers such as patient charges to obtain health services may have a greater impact on women with low income. Immigrant women’s life situation such as taking care of the elderly and children, language barriers in the new host country, and lack of knowledge of cancer and screening programs might also prevent them from participating in screening programs (Grandahl *et al.*, 2012). In our study, the association between being married and screening attendance varied for the different immigrant groups. Unmarried women from Eastern Europe, Asia, and South America took more Pap smear than married women from the same areas. Most of the previous studies showed that younger women take more Pap smears than older women, but information on marital status and Pap smear had been scarce. One report from British Columbia showed a positive association between being married and Pap smear for immigrants (Fletcher, 2011).

Our result showing that women in rural areas take more Pap smear was consistent for all groups. This is, to our knowledge, a new finding not described before. Immigrant women from rural areas tend to be better integrated into society and rural GPs have lower numbers of patients. As a result, information on and availability of the system might be higher.

Structural barriers include those related to physicians and the availability of the health system in the host country. Among the GP characteristics in our study, the main factor that was positively associated with Pap smear was having a female GP. There are other studies that show similar findings both related to women’s preferences (Nguyen *et al.*, 2002), but also to female GPs more actively asking new patients whether they have had a Pap smear (Harcourt *et al.*, 2014). This may also be the case in Norway. A recommendation by the GP has been described previously as an important facilitator to cancer screening (de Alba and Sweningson, 2006). Our study points to a lower screening attendance among women who have a GP with an immigrant background. This is in agreement with other studies suggesting that when the physician and the patient have the same immigrant background or ethnicity, the rate of CCS is reduced (McPhee *et al.*, 1997). In addition, lack of time to discuss screening and to communicate with the patient in a culturally appropriate way are mechanisms described to explain the low rate of CCS among immigrants (de Alba and Sweningson, 2006; Akers *et al.*, 2007).

### Strength and limitations of the study

Our study has several strengths. First, it is register based and includes over one million women. By including all the women registered in 2008 as having had a Pap smear, we avoid self-selection bias and by using GPs registration of tests, recall bias or errors with respect to diagnosis are minimal. Furthermore, grouping immigrant women by major world regions, we disentangled some of the differences between immigrant groups. Patterns observed among different immigrant groups in Norway are likely to be applicable to other Western countries.

However, our study also has limitations. The world regions that we use can be quite heterogeneous as they include many countries, religions, and cultures. GPs have a gatekeeper function in Norway and they take most of the Pap smears, but Pap smears taken by gynecologists or other health providers were not included in our data. However, women cannot seek a public gynecologist without a referral from a GP. Because we are using HELFO’s diagnosis system, we are dependent on GPs registering the Pap smears correctly. Some women might not be registered if they visit their GP for other reasons even though the consultation resulted in taking a Pap smear. For example, when a woman comes to see her GP for irregular bleeding, the diagnosis of menorrhagia is made even though the GP takes a Pap smear. Last but not the least, screening in Norway is recommended every 3 years, whereas we have studied Pap smear for only 1 year (2008). The lack of registration when several diagnoses are discussed in the consultation is probably the main reason for the discrepancy between our numbers (7.7% in 2008) and the ∼64% (around 20% per year) of women who take a Pap smear in a given year. However, on the basis of several other studies using HELFO data, there is no indication that GP’s registration is different for immigrants and nonimmigrants. Thus, we believe that these shortcomings will not change our results as our aim is not to determine the prevalence, but to compare the proportion of screening among nonimmigrants and immigrants.

### Implication for clinical practice

Our findings indicate the need for policy makers to develop and implement measures targeting the prevention of cervical cancer among immigrants. Increased awareness among primary care providers of low attendance among immigrants is required to increase participation of immigrants to preventive programs. GPs and other health providers need to know and learn more about barriers related to sex, communication, and culture to address these in an appropriate way.

### Conclusion

The participation of immigrant women to CCS in Norway must be increased. Appropriate interventions targeting both immigrant women and care providers need to be developed and evaluated. User participation and seeking information from immigrant women and healthcare personnel could further shed light on potential barriers and to decrease the screening gap between immigrants and nonimmigrants.

## Supplementary Material

SUPPLEMENTARY MATERIAL

Supplemental digital content is available for this article. Direct URL citations appear in the printed text and are provided in the HTML and PDF versions of this article on the journal's website (*www.eurjcancerprev.com*).

## References

[R1] Akers AY Newmann SJ Smith JS (2007). Factors underlying disparities in cervical cancer incidence, screening, and treatment in the United States. Curr Probl Cancer 31:157–181. 17543946 10.1016/j.currproblcancer.2007.01.001

[R2] Arnold M Razum O Coebergh J-W (2010). Cancer risk diversity in non-western migrants to Europe: an overview of the literature. Eur J Cancer 46:2647–2659. 20843493 10.1016/j.ejca.2010.07.050

[R3] Azerkan F Sparén P Sandin S Tillgren P Faxelid E Zendehdel K (2012). Cervical screening participation and risk among Swedish‐born and immigrant women in Sweden. Int J Cancer 130:937–947. 21437898 10.1002/ijc.26084

[R4] Berens E-M Stahl L Yilmaz-Aslan Y Sauzet O Spallek J Razum O Berens E-M (2014). Participation in breast cancer screening among women of Turkish origin in Germany – a register-based study. BMC Womens Health 14:24–24. 24507093 10.1186/1472-6874-14-24 PMC3922307

[R5] Campari C Fedato C Iossa A Petrelli A Zorzi M Anghinoni E (2015). Cervical cancer screening in immigrant women in Italy: a survey on participation, cytology and histology results. Eur J Cancer Prev 25:321–328. 10.1097/CEJ.0000000000000173 26207563

[R6] Cancer Registry of Norway (2016). Cervix cancer. Oslo: Cancer Registry of Norway.

[R7] Coughlin SS King J Richards TB Ekwueme DU (2006). Cervical cancer screening among women in metropolitan areas of the United States by individual-level and area-based measures of socioeconomic status, 2000 to 2002. Cancer Epidemiol Biomarkers Prev 15:2154–2159. 17119040 10.1158/1055-9965.EPI-05-0914

[R8] De Alba I Sweningson JM (2006). English proficiency and physicians’ recommendation of Pap smears among Hispanics. Cancer Detect Prev 30:292–296. 16844320 10.1016/j.cdp.2006.05.003

[R9] Echeverria ES Carrasquillo EO (2006). The roles of citizenship status, acculturation, and health insurance in breast and cervical cancer screening among immigrant women. Med Care 44:788–792. 16862042 10.1097/01.mlr.0000215863.24214.41

[R10] Ekechi C Olaitan A Ellis R Koris J Amajuoyi A Marlow LAV (2014). Knowledge of cervical cancer and attendance at cervical cancer screening: a survey of Black women in London. BMC Public Health 14:1096. 25339243 10.1186/1471-2458-14-1096 PMC4216339

[R11] Ferlay J Steliarova-Foucher E Lortet-Tieulent J Rosso S Coebergh JW Comber H (2013). Cancer incidence and mortality patterns in Europe: estimates for 40 countries in 2012. Eur J Cancer 49:1374–1403. 23485231 10.1016/j.ejca.2012.12.027

[R12] Fletcher JL Cervical cancer screening in immigrant populations in British Columbia : participation rates and sociodemographic characteristics of use [electronic theses and dissertations]. Vancouver, BC: University of British Columbia Library, British Columbia; 2011.

[R13] Ghebre R Sewali B Osman S Adawe A Nguyen H Okuyemi K Joseph A (2015). Cervical cancer: barriers to screening in the somali community in Minnesota. J Immigr Minor Health 17:722–728. 25073605 10.1007/s10903-014-0080-1 PMC4312274

[R14] Grandahl M Tyden T Gottvall M Westerling R Oscarsson M (2012). Immigrant women’s experiences and views on the prevention of cervical cancer: a qualitative study. Health Expect 18:344–354. 23252449 10.1111/hex.12034 PMC5060783

[R15] Harcourt N Ghebre R Whembolua G-L Zhang Y Warfa Osman S Okuyemi K (2014). Factors associated with breast and cervical cancer screening behavior among african immigrant women in Minnesota. J Immigr Minor Health 16:450–456. 23334709 10.1007/s10903-012-9766-4 PMC3644538

[R16] Jemal A Bray F Center MM Ferlay J Ward E Forman D (2011). Global cancer statistics. CA Cancer J Clin 61:69–90. 21296855 10.3322/caac.20107

[R17] Johnson CE Mues KE Mayne SL Kiblawi AN (2008). Cervical cancer screening among immigrants and ethnic minorities: a systematic review using the Health Belief Model. J Low Genit Tract Dis 12:232–241. 18596467 10.1097/LGT.0b013e31815d8d88

[R18] Lamkaddem M Spreeuwenberg PM Devillé WL Foets MM Groenewegen PP (2012). Importance of quality aspects of GP care among ethnic minorities: role of cultural attitudes, language and healthcare system of reference. Scand J Public Health 40:25–34. 22013158 10.1177/1403494811425710

[R19] Lee H Yang P Lee D Ghebre R (2015). Cervical cancer screening behavior among hmong-american immigrant women. Am J Health Behav 39:301–307. 25741675 10.5993/AJHB.39.3.2

[R20] Lofters A Moineddin R Hwang S Glazier RH (2010). Low rates of cervical cancer screening among urban immigrants a population-based study in Ontario, Canada. Med Care 48:611–618. 20548258 10.1097/MLR.0b013e3181d6886f

[R21] Lofters AK Moineddin R Hwang SW Glazier RH (2011). Predictors of low cervical cancer screening among immigrant women in Ontario, Canada. BMC Womens Health 11:20. 21619609 10.1186/1472-6874-11-20 PMC3121675

[R22] McDonald J Kennedy S (2007). Cervical cancer screening by immigrant and minority women in Canada. J Immigr Minor Health 9:323–334. 17345152 10.1007/s10903-007-9046-x

[R23] McPhee SJ Bird JA Davis T Ha NT Jenkins CN Le B (1997). Barriers to breast and cervical cancer screening among Vietnamese–American women. Am J Prev Med 13:205–213. 9181209

[R24] Nguyen TT McPhee SJ Nguyen T Lam T Mock J (2002). Predictors of cervical Pap smear screening awareness, intention, and receipt among Vietnamese–American women. Am J Prev Med 23:207–214. 12350454 10.1016/s0749-3797(02)00499-3 PMC1592337

[R25] Schleicher E (2007). Immigrant women and cervical cancer prevention in the United States. Baltimore, MD: Womem’s and Children’s Health Policy Center, Johns Hopkins Bloomberg School of Public Health.

[R26] Shelton RC Jandorf L Thelemaque L King S Erwin DO (2012). Sociocultural determinants of breast and cervical cancer screening adherence: an examination of variation among immigrant latinas by country of origin. J Health Care Poor Underserved 23:1768–1792. 23698689 10.1353/hpu.2012.0191

[R27] Skare GB Lönnberg S Cervical cancer screening. Annual report 2013–2014. (In Norwegian). Oslo, Norway; 2015.

[R28] Statistics Norway (2016). Immigrants and Norwegian-born to immigrant parents, 1 January 2016, StatBank, Table 07108. Oslo: Statistics Norway.

[R29] Woltman KJ Newbold KB (2007). Immigrant women and cervical cancer screening uptake: a multilevel analysis. Can J Public Health 98:470–475. 19039885 10.1007/BF03405441 PMC6975610

