# Supplementary material for: Differences in cervical cancer screening between immigrants and nonimmigrants in Norway: a primary healthcare register-based study
Source: Eur J Cancer Prev. 2017 Oct 5;26(6):521–7. doi: 10.1097/CEJ.0000000000000311 (PMC5627531; doi:10.1097/CEJ.0000000000000311)
Supplement: SUPPLEMENTARY MATERIAL [file cej-26-521-s001.docx]

**Supplementary Table 1: Socio demographic characteristics between women who have taken a Pap smear (P+) in 2008 and those who haven’t (P-)**

|  |  | | | | | | | | | | | |
| --- | --- | --- | --- | --- | --- | --- | --- | --- | --- | --- | --- | --- |
|  | **Norway** | | **Western Europe^*^** | | **Eastern Europe** | | **Asia** | | **Africa** | | **South America**** | |
|  | P+ | P- | P+ | P- | P+ | P- | P+ | P- | P+ | P- | P+ | P- |
| **Age** | 45.5 | 47.3 | 43.8 | 45.2 | 38.4 | 39.2 | 39.2 | 39.5 | 36.7 | 37.2 | 39.2 | 40.2 |
| St.dev. | 12.2 | 12.6 | 12.2 | 12.8 | 9.9 | 10.9 | 9.0 | 10.5 | 8.8 | 9.3 | 10.2 | 10.8 |
| **Length of stay** | - | - | 16.9 | 17.5 | 9.3 | 8.5 | 12.1 | 12.0 | 9.4 | 9.5 | 12.2 | 12.5 |
| St.dev. | - | - | 14.1 | 14.9 | 8.1 | 8.6 | 8.9 | 9.5 | 8.6 | 8.4 | 9.8 | 10.1 |
| **Income** |  |  |  |  |  |  |  |  |  |  |  |  |
| Low | 33.1 | 38.9 | 34.3 | 41.9 | 44.7 | 53.6 | 58.0 | 64.8 | 67.0 | 72.8 | 51.9 | 57.0 |
| Medium | 46.2 | 41.8 | 41.8 | 34.8 | 44.2 | 35.4 | 33.8 | 27.9 | 27.9 | 22.1 | 38.7 | 33.7 |
| High | 20.6 | 19.3 | 23.8 | 23.3 | 11.1 | 11.0 | 8.2 | 7.3 | 5.2 | 5.2 | 9.4 | 9.3 |
| **Education** |  |  |  |  |  |  |  |  |  |  |  |  |
| No | 0 | 0.1 | 0.4 | 0.3 | 0.4 | 0.8 | 3.4 | 5.2 | 7.4 | 9.6 | 0.7 | 1.5 |
| Low | 18.8 | 22 | 12.5 | 14.2 | 26.9 | 26.8 | 44.2 | 42.9 | 41.8 | 46.4 | 28.4 | 29.6 |
| Middle | 43.1 | 43.2 | 28.3 | 28.8 | 30.5 | 30.3 | 25.4 | 25.2 | 30.2 | 26.5 | 35.3 | 34.8 |
| High | 38.0 | 34.7 | 58.9 | 56.6 | 42.3 | 42.2 | 27.0 | 26.7 | 20.7 | 17.5 | 35.6 | 34.1 |
| **Municip. centr.** |  |  |  |  |  |  |  |  |  |  |  |  |
| Rural | 38.3 | 34.0 | 25.1 | 22.8 | 27.8 | 24.8 | 17.7 | 15.6 | 18.0 | 15.0 | 20.0 | 17.5 |
| Urban | 61.7 | 66 | 74.9 | 77.2 | 72.2 | 75.2 | 82.3 | 84.4 | 82.0 | 85.0 | 80.0 | 82.5 |
| **Marital status** |  |  |  |  |  |  |  |  |  |  |  |  |
| Married | 28.0 | 27.2 | 29.1 | 28.8 | 15.3 | 18.1 | 5.7 | 10.5 | 17.4 | 17.9 | 11.7 | 15.2 |
| Unmarried | 54.5 | 53.3 | 56.2 | 54.6 | 67.2 | 64.8 | 77.9 | 72.1 | 58.4 | 55.1 | 62.7 | 61.1 |
| Other | 17.5 | 19.5 | 14.7 | 16.6 | 17.4 | 17.0 | 16.4 | 17.4 | 24.3 | 27.1 | 25.6 | 23.7 |
| **Birth in 2008/09** | 4.9 | 5.8 | 7.1 | 7.7 | 9.9 | 9.8 | 9.1 | 10.2 | 16.7 | 17.6 | 8.8 | 10.0 |
| **Number of obs.** | 1168832 | | 40761 | | 35046 | | 55866 | | 14008 | | 7119 | |

*Western Europe includes also Nordic countries and North America.

**South America includes Central America
