# Supplementary material for: Differences in cervical cancer screening between immigrants and nonimmigrants in Norway: a primary healthcare register-based study
Source: Eur J Cancer Prev. 2017 Oct 5;26(6):521–7. doi: 10.1097/CEJ.0000000000000311 (PMC5627531; doi:10.1097/CEJ.0000000000000311)
Supplement: SUPPLEMENTARY MATERIAL [file cej-26-521-s002.docx]

**Supplementary table 2: Association between length of stay in Norway and Pap smear attendance**

| **Stay in Norway** | **Western Europe*** | | **Eastern Europe** | | **Asia** | | **Africa** | | **South America**** | |
| --- | --- | --- | --- | --- | --- | --- | --- | --- | --- | --- |
|  | OR | 95% CI | OR | 95% CI | OR | 95% CI | OR | 95% CI | OR | 95% CI |
| 0-2 yrs | 1 |  | 1 |  | 1 |  | 1 |  | 1 |  |
| 3 yrs + | 0.86 | 0.76-0.98 | 1.17 | 1.02-1.33 | 0.93 | 0.82-1.04 | 0.74 | 0.59-0.92 | 0.74 | 0.56-0.98 |
| 0-5 yrs | 1 |  | 1 |  | 1 |  | 1 |  | 1 |  |
| 6 yrs + | 0.93 | 0.83-1.03 | 1.13 | 1.01-1.27 | 1.02 | 0.93-1.11 | 0.75 | 0.63-0.90 | 0.95 | 0.75-1.21 |
| 0-10 yrs | 1 |  | 1 |  | 1 |  | 1 |  | 1 |  |
| 11 yrs + | 0.98 | 0.88-1.09 | 1.13 | 1.00-1.27 | 0.99 | 0.91-1.07 | 0.92 | 0.76-1.11 | 1.01 | 0.80-1.28 |
| 0-15 yrs | 1 |  | 1 |  | 1 |  | 1 |  | 1 |  |
| 16 yrs + | 1.06 | 0.95-1.18 | 1.01 | 0.87-1.18 | 0.95 | 0.87-1.03 | 0.98 | 0.79-1.20 | 1.03 | 0.81-1.30 |
| 0-20 yrs | 1 |  | 1 |  | 1 |  | 1 |  | 1 |  |
| 21 yrs + | 0.97 | 0.86-1.08 | 1.05 | 0.85-1.30 | 0.96 | 0.87-1.06 | 1.13 | 0.85-1.49 | 0.97 | 0.74-1.21 |

*Western Europe includes also Nordic countries and North America.

**South America includes Central America
